# Digital pathology and artificial intelligence in breast and gynecologic oncology: from molecular prediction to multimodal integration

**DOI:** 10.3389/fonc.2026.1833926

**Published:** 2026-04-24

**Authors:** Francesca Polit, Hisham F. Bahmad, Mohamad B. Kassab, Mohamad K. Elajami, Monica Recine, Sarah Alghamdi, Robert Poppiti

**Affiliations:** 1 Arkadi M. Rywlin M.D. Department of Pathology and Laboratory Medicine, Mount Sinai Medical Center of Florida, Miami Beach, FL, United States; 2 Department of Pathology and Laboratory Medicine, University of Miami Miller School of Medicine, Miami, FL, United States; 3 Cardiovascular Research Center, Massachusetts General Hospital, Boston, MA, United States; 4 Department of Internal Medicine, Hartford Hospital, Hartford, CT, United States; 5 Herbert Wertheim College of Medicine, Florida International University, Miami, FL, United States; 6 Seymon and Janna Advanced Research Institute at Mount Sinai Medical Center, Miami Beach, FL, United States

**Keywords:** artificial intelligence, breast cancer, digital pathology, gynecologic oncology, machine learning, molecular pathology, multimodal integration, precision medicine

## Abstract

Breast and gynecologic cancers consist of two groups of complex solid tumors, each with unique genomic features, immune microenvironments, and treatment responses. Recent advances in next-generation sequencing, spatial profiling, and digital pathology have transformed diagnostic methods, enabling seamless integration of morphological and molecular data. Artificial intelligence (AI) and machine learning (ML) are now essential tools for linking histomorphology, immunophenotype, and molecular alterations in ways that were previously unachievable. This review discusses recent progress in integrating digital and molecular pathology for these cancers, with an emphasis on practical clinical applications. We highlight emerging research in breast, endometrial, ovarian, and cervical cancers, where combined image-based and molecular approaches can predict treatment response and survival. Additionally, spatial transcriptomics and proteomics are deepening our understanding of tumor heterogeneity and the interactions between tumor cells, stroma, and immune cells that drive disease progression. We also address current challenges, such as standardization, reproducibility, regulation, and workflow integration, and propose priorities to facilitate the clinical adoption of multimodal data.

## 1 Introduction

Pathology has long acted as the interpretive link between tissue structure and disease biology, but in breast and gynecologic oncology, this role has expanded quickly. Tumors once primarily identified through pattern recognition are now classified based on molecular drivers, immune contexture, therapeutic targets, and their spatial arrangement within the tumor microenvironment (TME) (1). While molecular pathology has provided unprecedented insights into tumor biology and facilitated the development of targeted therapies, it has also introduced significant complexity. The increased costs, tissue requirements, longer turnaround times, and workflow fragmentation can restrict both widespread access and consistent implementation (2, 3). Meanwhile, digital pathology has progressed far beyond its initial roles in archiving and teleconsultation. It has become a quantitative platform increasingly capable of capturing morphologic signals that relate to genomics, prognosis, and treatment response, especially when integrated with modern artificial intelligence (AI) methods and machine learning (ML) (4, 5).

In routine clinical practice, molecular testing, spatial profiling, and image-based analysis are often performed separately, producing independent results and reports, with critical information remaining isolated and difficult to combine at the point of care. This gap has serious clinical consequences because breast and gynecologic cancers are biologically complex, and management decisions frequently depend on a multidisciplinary, holistic approach that requires integrating multiple layers of evidence for optimal patient care.

Breast cancer illustrates the molecular revolution of the late 20th and early 21st centuries, where routine classification now includes hormone receptor status, HER2 amplification, and multigene expression assays that guide both prognosis and treatment choices (6, 7). Similarly, endometrial carcinomas are now classified using integrated molecular frameworks that include mismatch repair status, *POLE* mutations, *p53* abnormalities, and copy number profiles. This approach has fundamentally reshaped risk stratification and treatment (8, 9). For instance, high-grade serous ovarian carcinoma (HGSOC) is known as a primarily *TP53*-driven disease with links to homologous recombination deficiency and *BRCA1/2* status, while cervical carcinomas are increasingly characterized by HPV-related molecular pathways and immune checkpoint expression patterns (10–12).

Digital pathology, originally used for slide archiving, remote consultation, and education, has evolved into a quantitative field capable of extracting biologically meaningful features from routine hematoxylin and eosin (H&E) slides that relate to genomic alterations, prognosis, and treatment response (13, 14). The U.S. Food and Drug Administration’s (FDA’s) regulatory approval of whole-slide imaging (WSI) systems for primary diagnosis in 2017 (granted to the Philips IntelliSite Pathology Solution) removed a major barrier to clinical deployment, enabling broader adoption across academic medical centers and community practices (13, 15–17). Parallel advances in scanner fidelity, image compression algorithms, cloud storage infrastructure, and laboratory information system integration have further sped up this digital transformation.

The shift from glass slides viewed under optical microscopes to digital Whole Slide Images (WSIs) marks a major change in pathology practice. Early digital pathology efforts primarily focused on improving operational efficiency, including remote consultations, multidisciplinary tumor board reviews, educational uses, and archival storage (18). Digitization, however, also facilitated quantitative image analysis, laying crucial groundwork for computational pathology and AI-driven diagnostics (19). Initial computational algorithms focused on well-defined, relatively simple tasks such as automated mitotic figure counting, measurement of the Ki-67 proliferation index, and immunohistochemical (IHC) scoring of hormone receptors and HER2 (20). These early systems relied predominantly on rule-based approaches and hand-crafted features that required extensive domain expertise to develop and often struggled with the biological variability inherent in clinical specimens.

The emergence of AI and ML, particularly deep learning (DL) architectures based on convolutional neural networks (CNNs) and, more recently, vision transformers, has accelerated this evolution by enabling automated detection of complex morphologic patterns previously imperceptible to human observers or too subtle for traditional rule-based image analysis (8, 21). These AI models can simultaneously analyze thousands of morphological features, capturing subtle patterns in nuclear morphology (size, shape, and chromatin pattern), tissue architecture, stromal composition, and immune infiltration, to generate data-driven algorithmic models that are capable of predicting molecular alterations, treatment response, and clinical outcomes (4, 22). Recent advances include the development of foundation models (large-scale AI models pretrained on millions of histopathology images from diverse tissue types and disease states) that can be efficiently fine-tuned for specific diagnostic tasks with relatively small datasets. These foundation models have demonstrated superior performance compared to models trained from scratch, particularly in scenarios with limited annotated training data, and show improved generalization across different institutions, scanners, and staining protocols.

Campanella et al. demonstrated the real-world deployment of a fine-tuned pathology foundation model for lung cancer biomarker detection, providing one of the first clinically validated examples of foundation model-based AI triage in routine pathology practice (23). Nevertheless, while AI-driven computational pathology offers objective and reproducible analytical capabilities, it should be used only as a decision-support tool that enhances, rather than replaces, the clinical judgment and contextual expertise of pathologists (24, 25). The integration of digital and molecular pathology provides an opportunity to develop synergistic workflows where AI models trained on histomorphology can predict molecular features, triage cases for confirmatory testing, and discover new morphological-molecular correlations that enhance our understanding of cancer biology (26–28).

The development of spatial profiling technologies, including spatial transcriptomics, spatial proteomics, and multiplexed imaging platforms, has introduced a vital spatial dimension to molecular pathology, enabling gene and protein expression to be mapped within intact tissue structures (29–31). These methods have uncovered previously hidden aspects of intratumoral heterogeneity, immune-tumor interactions, and stromal signaling networks that affect treatment response and disease progression (32, 33). Digital pathology offers the crucial visual basis for interpreting spatial molecular data, with AI-assisted cell segmentation, phenotyping, and spatial analysis allowing scalable examination of these complex datasets (34, 35). It is important to note that these spatial profiling technologies, while highly informative for research, are not yet part of routine clinical practice and remain largely restricted to research use, with clinical translation still in early stages.

This review examines how the integration of digital pathology, molecular profiling, and AI/ML is transforming practice in breast and gynecologic oncology. We highlight clinically relevant advances validated by multiple studies, discuss biological insights from multimodal data integration, explore current implementation challenges, including regulatory issues and workflow integration, and outline future directions that place pathologists at the heart of precision medicine efforts. By synthesizing evidence from recent research and clinical trials, we offer a comprehensive roadmap for applying these converging technologies to enhance patient care.

## 2 Molecular pathology in breast and gynecologic oncology

### 2.1 Breast cancer: a molecularly stratified disease

Breast cancer exemplifies the clinical value and complexity of molecular stratification in oncology. Hormone receptor status (estrogen receptor (ER) and progesterone receptor (PR)), *HER2* amplification, and multigene expression assays including Oncotype DX, MammaPrint, and Prosigna guide therapeutic selection and prognostic assessment (36–39). More recently, somatic mutations in *ESR1*, *PIK3CA*, *AKT1*, and *BRCA1/2*, along with tumor mutational burden and PD-L1 expression, have become integral to therapeutic decision-making, particularly in advanced and metastatic disease (40–42).

Despite this progress, molecular testing remains resource-intensive, often requiring separate tissue sections, specialized platforms, considerable turnaround time, and substantial cost. Moreover, molecular assays typically lack spatial context, providing aggregate information about tumor biology without capturing intratumoral heterogeneity or the spatial organization of molecular features within tissue architecture (43, 44). This gap between molecular profiling and histologic context limits real-time integration at the diagnostic bench and may obscure clinically relevant spatial patterns of biomarker expression.

### 2.2 Gynecologic malignancies: molecular reclassification and therapeutic implications

Gynecologic oncology has undergone a similar molecular transformation. Endometrial carcinomas are now routinely classified using molecular frameworks that incorporate mismatch repair protein expression (or microsatellite instability testing), *POLE* exonuclease domain mutations, and p53 immunohistochemistry as a surrogate for TP53 mutation status (45). This molecular classification, endorsed by major professional societies including the NCCN, identifies four prognostically distinct subgroups: *POLE*-mutated (ultramutated, excellent prognosis), mismatch repair deficient (hypermutated, intermediate prognosis), *p53*-abnormal (copy number high, poor prognosis), and no specific molecular profile (NSMP, intermediate prognosis) (45, 46). These molecular classifications highlight the critical need for tools that can contextualize molecular findings within tumor architecture, capture spatial heterogeneity, and integrate morphological and molecular information in clinically meaningful ways.

### 2.3 Inferring genomic alterations from H&E slides in breast cancer

The ability to predict molecular biomarker status directly from routine H&E-stained slides offers several potential advantages over traditional molecular testing, including reduced costs, faster turnaround times, preservation of limited tissue for additional testing, and the capacity to map spatial heterogeneity of biomarker expression across the entire tumor. Multiple studies have demonstrated that DL models can predict critical molecular biomarkers directly from routine H&E WSIs with clinically relevant accuracy.

For ER status, AI models have achieved AUC values ranging from 0.89 to 0.97 across independent validation cohorts, with some studies reporting accuracies exceeding 90%; i.e., performance levels approaching the inter-observer reproducibility of conventional IHC interpretation (9, 47–49). In a landmark study, Naik et al. developed a multiple instance learning-based deep neural network that determined ER status from H&E WSIs with an AUC of 0.92 on a multi-country dataset of 3,474 patients, demonstrating generalization across diverse populations, different institutions, and multiple scanning platforms (9).

Prediction of progesterone receptor (PR) status has proven more challenging, with reported AUC values ranging from 0.81 to 0.88, reflecting the greater biological heterogeneity of PR expression patterns and the known variability in PR staining quality (7, 47). HER2 status prediction from H&E images has achieved AUC values between 0.70 and 0.90 in most studies, with performance varying based on the complexity of the classification task (binary HER2-positive vs. HER2-negative vs. multiclass scoring) (49–52). Notably, Bychkov et al. demonstrated that morphology-based HER2 predictions not only correlated with gene amplification status but also predicted trastuzumab treatment efficacy, with patients having higher AI-derived HER2 scores showing more favorable distant disease-free survival (HR 0.37, 95% CI 0.15-0.93, *P* = 0.034) (51).

Recent advances include cross-modality learning approaches that leverage paired H&E and IHC images during training to improve prediction accuracy. Das et al. developed HistoStainAlign, a contrastive learning framework that achieved weighted F1 scores of 0.735 for P53, 0.830 for PD-L1, and 0.723 for Ki-67 prediction from H&E slides, demonstrating the potential for pre-screening applications that could prioritize cases for confirmatory IHC testing and optimize laboratory workflows (53).

With respect to molecular subtyping of breast cancer, AI models have successfully predicted intrinsic subtypes from H&E images. The pathomics breast cancer hierarchical image pyramid transformer (PBC-HIPT) model, utilizing a hierarchical image pyramid transformer architecture, achieved mean accuracy of 84.3% and mean AUC of 0.91 for three-class molecular subtyping (luminal, HER2-enriched, triple-negative) on tissue microarrays, with particularly strong performance for ER status (91.8% accuracy, AUC 0.97) and PR status (92.0% accuracy, AUC 0.96) (54). Another validation study on a population-based cohort demonstrated strong performance in distinguishing triple-negative breast cancer (TNBC) from non-TNBC subtypes (AUC 0.823, accuracy 0.833), though performance declined with increasing number of subtype classes, highlighting the ongoing challenge of fine-grained molecular classification (48).

Importantly, several studies have demonstrated that AI-predicted molecular subtypes can capture intratumoral heterogeneity that may be missed by single-site tissue sampling or bulk molecular assays performed on limited biopsy material. This spatial resolution represents a significant advantage over conventional approaches, as patients showing heterogeneous subtype predictions across different tumor regions have been shown to exhibit intermediate survival outcomes between homogeneous subtype groups, suggesting that AI may identify clinically meaningful biological diversity that impacts prognosis (55). This capability to map biomarker heterogeneity across entire surgical specimens could inform more comprehensive tumor characterization and potentially guide sampling strategies for molecular testing.

Emerging research has extended beyond individual biomarkers to predict broader gene expression patterns from histomorphology. Wang et al. performed the first transcriptome-wide expression-morphology analysis in breast cancer, training individual deep convolutional neural networks for 17,695 genes and achieving significant associations between predicted and actual RNA sequencing estimates for 9,334 genes (52.75%) (56). A newer, computationally efficient approach called hist2RNA, inspired by bulk RNA sequencing techniques, demonstrated successful prediction of 138 genes from six commercial molecular profiling tests, achieving correlation of 0.82 across patients and showing prognostic significance for overall survival independent of standard clinicopathological variables (c-index 0.65, HR 1.87, P<0.005) (12). These transcriptome-wide approaches enable prediction of proliferation scores, immune signatures, and pathway activities directly from routine histology, providing spatial resolution of gene expression patterns within the tumor while potentially reducing reliance on expensive molecular testing in selected clinical contexts (56).

Several recent studies have further advanced WSI-based gene expression prediction using novel computational frameworks. SEQUOIA, a linearized transformer model developed by Pizurica et al., predicts cancer transcriptomic profiles from WSIs across 16 cancer types using 7,584 tumor samples, with generalization validated on two independent cohorts comprising 1,368 tumors; accurately predicted genes were associated with key cancer processes including inflammatory response, cell cycle regulation, and metabolism, and the model demonstrated clinical utility in stratifying breast cancer recurrence risk and resolving spatial gene expression at loco-regional levels (57). DeepPT introduced a three-component deep learning architecture (CNN feature extraction, autoencoder compression, and multi-layer perceptron regression) for imputing gene expression from H&E slides. The model, often used in conjunction with the ENLIGHT framework (as part of the ENLIGHT-DeepPT pipeline), has been validated across multiple independent clinical datasets to predict cancer treatment response (58). STimage presented a probabilistic deep learning framework that prioritizes robustness and interpretability for spatial transcriptomics prediction from histopathology images, demonstrating that predicted gene expression can stratify patient survival and predict drug response (59). Additional approaches include HGGEP, which employs hypergraph neural networks to capture higher-order associations among multiple latent-stage features from WSIs for improved gene expression prediction, and PEKA (60, 61), a knowledge transfer framework that bridges pathology and single-cell foundation models to enhance gene expression prediction accuracy by aligning histopathology image embeddings with transcriptomic domain knowledge. Collectively, these advances demonstrate the rapidly maturing capability of predicting molecular profiles directly from routine histology, though further prospective validation is needed before clinical implementation (62).

AI models trained on H&E WSIs have demonstrated the capability to predict patient prognosis and survival outcomes across breast malignancies. The DiaDeepBreastPRS deep neural network model achieved a c-index of 67% for predicting 5-year overall survival on the TCGA-BRCA dataset, which increased to 78% when combined with pTNM stage and age at diagnosis (63). The AI-derived risk score was independently associated with survival (HR 2.46, P<0.005) and successfully stratified patients into good- and poor-prognosis groups (63).

Notably, Bidard et al. developed and externally validated a clinicopathologic assay integrating AI-derived histomorphological features with standard clinical variables to identify patients with low relapse rates despite having clinically high-risk ER-positive/HER2-negative early breast cancer (64). This represents one of the most extensively externally validated AI-based prognostic assays in breast cancer to date, demonstrating that AI-augmented clinicopathologic assessment can refine risk stratification beyond conventional tools and potentially spare low-risk patients from unnecessary adjuvant chemotherapy.

Quantitative digital histopathology features, particularly graph-based and wavelet features extracted from pre-treatment tumor biopsies, have also shown promise in predicting pathological complete response (pCR) to neoadjuvant chemotherapy in breast cancer. A gradient boosting machine model incorporating these features achieved an AUC of 0.90, a sensitivity of 85%, and a specificity of 82% on an independent test set, substantially outperforming models based solely on clinical features (AUC 0.73) (65). For HER2-positive breast cancer, DL models trained on H&E tumor regions of interest predicted trastuzumab treatment response with an AUC of 0.80 in five-fold cross-validation, suggesting that morphological features capture biological determinants of anti-HER2 therapy efficacy (66). These findings suggest that AI-based histomorphological analysis could serve as a complementary decision support tool and, in resource-limited settings where molecular testing is unavailable, potentially help guide treatment selection.

### 2.4 Predicting molecular alterations from histomorphology in gynecologic malignancies

Molecular classification of endometrial cancer has become a cornerstone of contemporary risk stratification and treatment planning, as recognized by international guidelines from ESGO/ESTRO/ESP, which now recommend molecular profiling to guide adjuvant therapy decisions (67). The four molecular subgroups (*POLE*-mutated (POLE^mut^), mismatch repair-deficient (MMRd), p53-abnormal (p53abn), and no specific molecular profile (NSMP)) carry distinct prognostic implications and may influence therapeutic approaches (45, 67). AI-based prediction of these molecular subtypes directly from routine H&E slides offers the potential for rapid, cost-effective classification that could be performed on diagnostic biopsies to inform surgical planning and adjuvant treatment discussions, particularly in resource-limited settings where comprehensive molecular testing may not be readily available.

AI models have demonstrated strong performance in predicting the four molecular subgroups of endometrial cancer directly from H&E slides (3, 46, 68). The im4MEC model (interpretable deep learning model to predict the molecular classification of endometrial cancer), developed through combined analysis of the PORTEC randomized trials and clinical cohorts (n=2,028), achieved macro-average area under the receiver operating characteristic curve (AUROC) of 0.874 on four-fold cross-validation and 0.876 on an independent test set, with class-wise AUROCs of 0.849 for POLE^mut^, 0.844 for MMRd, 0.883 for NSMP, and 0.928 for p53abn (68). A recent study by Guo et al. developed another interpretable DL model that achieved macro-average AUROC of 0.867 (95% CI 0.823-0.911) in five-fold cross-validation, with class-wise AUROCs of 0.846 for MSI-H, 0.876 for NSMP, 0.910 for p53abn, and 0.835 for POLE^mut^ subtypes (3). These performance levels suggest potential clinical utility, though they remain below the near-perfect accuracy of definitive molecular testing and would likely function best as screening tools to prioritize cases for confirmatory analysis.

It should be emphasized that these AI-based approaches for predicting molecular subtypes from H&E slides are currently for research use only and have not yet been validated for routine clinical deployment. Prospective clinical trials and regulatory approval will be necessary before these tools can be integrated into standard diagnostic workflows.

Importantly, morphological analysis revealed distinct histologic features associated with each molecular subtype, aligning with pathologists’ observational experience. MSI-H tumors exhibited increased stromal lymphocytic infiltration, POLE^mut^ tumors showed higher heterogeneity and solid growth patterns, p53abn tumors demonstrated papillary architecture and serous-like features, while NSMP tumors displayed high stromal cellularity (3). These interpretable morphological signatures provide biological plausibility for the AI predictions and may enhance pathologist confidence in model outputs.

For predicting MMR deficiency or microsatellite instability status specifically, a finding with implications for both Lynch syndrome screening and immunotherapy eligibility, AI models have achieved AUC values reaching 0.82 in endometrial cancer (14, 69). A systematic review examining MSI prediction across multiple cancer types found that AI-based systems showed excellent performance in colorectal cancer (AUC up to 0.972) and satisfactory performance in endometrial cancer, suggesting potential utility as a pre-screening tool to triage cases for confirmatory immunohistochemistry or molecular testing and optimize laboratory workflows (14). The concordance between biopsy and hysterectomy specimens for molecular classification is high, with concordance rates of 97% for MMR status, 96% for *p53* status, and 99% for *POLE* mutations, supporting the feasibility of preoperative molecular subtyping using endometrial biopsy specimens for treatment planning (70). This high concordance is clinically significant as it enables AI models trained on hysterectomy specimens to be reliably applied to diagnostic biopsies, potentially allowing molecular risk stratification before definitive surgery.

AI-predicted molecular classifications have demonstrated prognostic significance beyond simple category assignment. The im4MEC model showed significant differences in 5-year recurrence-free survival between predicted molecular classes, with potential for prognostic refinement within molecular subgroups (68). Notably, patients with aggressive p53abn endometrial cancer predicted as MMRd showed inflammatory morphology and appeared to have better prognosis than correctly predicted p53abn cases, while NSMP cases predicted as p53abn showed higher nuclear atypia and worse outcomes (68). These findings suggest that morphology-based predictions may capture biologically relevant tumor features and microenvironmental characteristics beyond standard molecular classification, potentially identifying subsets of patients with intermediate biology who might benefit from different therapeutic approaches. However, these observations require validation in prospective studies before influencing clinical decision-making.

In ovarian cancer, particularly high-grade serous ovarian carcinoma (HGSOC), predicting platinum-based chemotherapy response remains a critical clinical challenge, as approximately 20-30% of patients exhibit primary platinum resistance (71–75). Ahn et al. developed a DL classifier that predicted platinum-based treatment responses in HGSOC with high accuracy, identifying morphological patterns associated with chemotherapy sensitivity, including features related to tumor-infiltrating lymphocytes (TILs) and stromal characteristics (74). Similarly, Wang et al. demonstrated that convolutional neural networks could predict treatment effectiveness from histopathology images with significant correlation to clinical outcomes (76). While these results are encouraging, it is important to note that most studies have been conducted in relatively small retrospective cohorts, and the remarkably high performance metrics warrant cautious interpretation pending larger, multi-institutional validation studies. If validated prospectively, such tools could potentially help identify patients who might benefit from alternative first-line therapeutic strategies or inclusion in clinical trials of novel agents.

## 3 Spatial profiling and the tumor microenvironment

Traditional bulk molecular profiling approaches provide averaged measurements across tissue samples, potentially obscuring critical spatial relationships between tumor cells, immune populations, and stromal components that influence disease progression and therapeutic response. Spatial profiling technologies address this limitation by preserving the geographic context of molecular measurements within intact tissue architecture, enabling characterization of the TME with unprecedented resolution. This spatial dimension is particularly relevant to pathologists, as it aligns with the visual assessment of tissue architecture routinely performed on H&E-stained slides and adds quantitative molecular data to morphological observations. Understanding the spatial organization of the TME has emerged as increasingly important for predicting immunotherapy response, identifying mechanisms of treatment resistance, and characterizing intratumoral heterogeneity that may not be captured by single-site biopsies.

### 3.1 Spatial profiling technologies

Current spatial profiling platforms fall into two main categories: spatial transcriptomics methods that measure gene expression, and spatial proteomics approaches that quantify protein abundance. Spatial transcriptomics platforms include imaging-based methods such as seqFISH, MERFISH, CosMx SMI, and Xenium, which offer subcellular resolution and can visualize individual RNA molecules within cells, and sequencing-based approaches like Visium and Stereo-seq, which provide broader tissue coverage by measuring gene expression in circular spots (each containing 1–10 cells) across entire tissue sections (77–80). Visium (10x Genomics) has emerged as the most widely adopted platform in cancer research due to its balance of tissue coverage, throughput, and cost, with SCTransform from the Seurat R library serving as the preferred method for data normalization and integration (78).

Spatial proteomics platforms, including GeoMx Digital Spatial Profiler (DSP), CODEX, Multi-Omyx, imaging mass spectrometry (IMS), and multiplex ion-beam imaging (MIBI), enable simultaneous measurement of dozens to hundreds of proteins while preserving spatial information within tissue sections (81, 82). These technologies complement spatial transcriptomics by providing direct measurement of protein abundance and post-translational modifications in their spatial context, offering insights into active signaling networks within the TME that cannot be inferred from RNA measurements alone (81, 82). The CosMx platform also offers protein profiling capabilities achieving up to 64-plex protein detection, bridging spatial transcriptomics and proteomics within a single platform and providing complementary protein-level spatial information alongside RNA measurements (83).

The integration of spatial transcriptomics with single-cell RNA sequencing has enabled computational deconvolution (the process of inferring the mixture of cell types present in each measurement spot) and reconstruction of cell type-specific gene expression profiles, revealing previously hidden aspects of intratumoral heterogeneity (84, 85). Spatial analysis has identified prognostic markers based on the spatial organization of immune and stromal cells, including the characterization of tertiary lymphoid structures (TLSs) and immune-excluded tumor regions where immune cells accumulate at tumor borders but fail to infiltrate, a pattern that correlates with resistance to immunotherapy (86).

### 3.2 Spatial profiling in breast cancer

In breast cancer, spatial transcriptomics has revealed multidimensional heterogeneity across molecular subtypes and histological variants, uncovering the dynamic interplay between tumor cells, immune cells, and stromal components that influences disease behavior (77, 84). Unlike traditional TILs scoring, which provides overall immune infiltration levels, spatial profiling has identified distinct TME niches (localized tissue regions with unique cellular compositions and molecular signatures) characterized by specific immune infiltration patterns, metabolic reprogramming, and stromal organization that influence therapeutic response and disease progression (77).

Studies have demonstrated that spatial patterns of TILs, cancer-associated fibroblast subtypes (which can be either tumor-promoting or tumor-restraining), and endothelial cell organization correlate with prognosis and treatment outcomes, with specific spatial configurations predicting response to chemotherapy, targeted therapy, and immunotherapy (77, 84). For example, the proximity of CD8+ T cells to tumor cells, rather than their absolute number, has emerged as a stronger predictor of immunotherapy efficacy. Therefore, future pathology assessment may need to incorporate not just “what cells are present” but “where they are located relative to each other,” a feature that spatial profiling technologies are beginning to enable at scale.

### 3.3 Spatial profiling in ovarian cancer

Spatial transcriptomics has proven particularly valuable in ovarian cancer, where it has revealed discrete tumor microenvironments and subclone-specific autocrine signaling loops (where tumor cells produce growth factors that stimulate their own proliferation) within individual tumor sections (87, 88). Denisenko et al. used Visium spatial transcriptomics to identify multiple tumor subclones with different copy number alterations present within individual tumor sections, demonstrating that these genetically distinct subclones differentially express various ligands and receptors and associate with distinct stromal and immune cell populations (88). Follow-up analysis using CosMx single-molecule imaging revealed that each subclone recruited specific immune cells, fibroblasts, and endothelial cells, with cell-to-cell communication analysis identifying subclone-specific signaling pathways and multiple autocrine loops (88). This finding has important implications for therapy, as it suggests that treating the “average” tumor biology may miss resistant subclones with fundamentally different dependencies.

Research on HGSOC has revealed distinct tumor-immune microenvironments associated with *BRCA1*/*2* mutation status and platinum response (89, 90). Launonen et al. used highly multiplex immunofluorescence to generate spatial proteomic data for 21 markers in 124,623 single cells from *BRCA1*/*2*-mutated tumors, identifying a phenotypically distinct TME with evidence of increased immunosurveillance and a prognostic proliferative tumor-cell subpopulation that associated with enhanced spatial tumor-immune interactions by CD8+ and CD4+ T cells (90). This suggests that *BRCA*-mutated tumors may be more immunologically “visible” and potentially more responsive to immunotherapy.

A spatial proteomic study comparing platinum-refractory and platinum-sensitive HGSOC using GeoMx DSP revealed critical differences in TME organization that help explain patterns of treatment resistance. Platinum-sensitive patients had elevated apoptotic markers and anti-tumor immune profiles, consistent with effective chemotherapy-induced cell death and immune engagement, while platinum-refractory patients exhibited dual activation of *AKT1* and *WNT* signaling pathways along with immunosuppressive profiles (89). Interestingly, the dual activity of *AKT1* and *WNT* signaling corresponded to the physical exclusion of TILs from the TME and abnormal endothelial cell structure, signifying that these tumors create a physical and biochemical barrier preventing immune cell infiltration (89). This spatial tumor-immune crosstalk represents a potentially targetable mechanism of resistance that would not be apparent from bulk molecular profiling.

Additional studies investigating the spatial distribution of gene expression in ovarian cancer have identified specific spatial features that impact prognosis and therapy outcomes, with evidence that spatial patterns of tumor cells and their immune and stromal microenvironment significantly influence chemotherapy response and disease progression (87). Spatial transcriptomics has illuminated mechanisms of immune evasion, metabolic adaptation, and stromal remodeling that occur in spatially distinct tumor regions, providing biological insights into treatment resistance that may inform the development of combination therapeutic strategies (87).

### 3.4 Spatial profiling in endometrial and cervical cancers

In endometrial cancer, spatial transcriptomics has been integrated with molecular classification frameworks to map the spatial distribution of POLE^mut^, MMRd, p53abn, and NSMP tumor regions within individual cases, revealing that some tumors exhibit intratumoral molecular heterogeneity (distinct molecular subtypes coexisting within the same specimen) that would be missed by single-site sampling (91). Spatial profiling has also identified patterns of immune infiltration associated with different molecular subtypes and characterized ligand-receptor interactions at the tumor-stroma interface that influence therapeutic response, with specific spatial configurations of immune cells correlating with immunotherapy sensitivity (81, 91). These findings raise important questions about whether molecular classification should account for spatial heterogeneity, particularly in cases where different regions might warrant different therapeutic approaches.

In cervical adenocarcinoma, Silva pattern-guided spatial proteomics (using laser-microdissection to separately analyze different architectural patterns) followed by quantitative proteomic analysis identified 7,300 proteins across tumor and stromal microcompartments (92). Silva pattern C tumors, which carry the worst prognosis, exhibited distinct proteomic signatures associated with aggressive tumor behavior, including upregulation of epithelial-mesenchymal transition markers and extracellular matrix remodeling proteins concentrated at the tumor-stroma interface (92).

### 3.5 Clinical translation: challenges and opportunities

The clinical translation of spatial profiling technologies faces several significant challenges. These include high cost (Visium analysis costs approximately USD 1,000-1,500 per sample), technical complexity requiring specialized laboratory infrastructure, limited throughput compared to routine histopathology (processing times of days to weeks versus hours), and the need for specialized bioinformatics expertise to analyze and interpret the resulting high-dimensional datasets (78, 79, 93). Additionally, standardization of protocols, quality control metrics, and pipeline analysis across institutions remains an ongoing challenge that must be addressed before widespread clinical adoption.

Despite these barriers, emerging applications demonstrate clinical utility that may justify investment in specific contexts. Spatial profiling shows particular promise in predicting immunotherapy response based on spatial patterns of PD-L1 expression (which can vary dramatically across tumor regions), the spatial organization and density of TILs relative to tumor cells, and the presence and location of TLSs (86). In the near term, spatial profiling may be most valuable for research applications that inform clinical trial design, biomarker development, and mechanistic understanding of treatment resistance, rather than routine diagnostic use. As technologies mature, become more cost-effective, and analytical workflows become standardized, selected spatial profiling assays may transition into clinical laboratories to complement conventional pathology assessment, particularly for challenging cases or treatment decisions where standard biomarkers provide insufficient guidance.

An important consideration for pathologists is that spatial profiling technologies generate data that complements rather than replaces morphological assessment. The integration of AI-based image analysis of H&E slides with spatial molecular profiling represents a particularly promising direction, as it could enable the prediction of spatial molecular features from routine histology, potentially democratizing access to spatial insights without requiring expensive specialized platforms for every case.

## 4 Foundation models and multimodal AI

### 4.1 The evolution toward foundation models

The field of AI in pathology is experiencing a fundamental shift from narrow, task-specific algorithms, each trained to perform a single function like detecting metastases or grading tumors, toward versatile foundation models that can adapt to multiple diagnostic tasks with minimal additional training (94, 95). This paradigm mirrors the evolution seen in natural language processing, where models like GPT demonstrated that large-scale pre-training on diverse data enables rapid adaptation to new tasks.

Foundation models in pathology, such as UNI, CONCH, GigaPath, mSTAR, and Atlas, are trained on millions of whole slide images from diverse tissue types, institutions, and scanning platforms using self-supervised learning, a technique that allows models to learn meaningful patterns from unlabeled images without requiring pathologist annotations for every training sample (94, 95). This approach addresses one of the major bottlenecks in AI development: the enormous cost and time required to obtain expert-labeled training data. Once pre-trained, these foundation models can be rapidly adapted to new tasks (such as predicting a novel biomarker or classifying a rare tumor subtype) with far fewer labeled examples than would be required to train a model from scratch.

These models demonstrate remarkable capabilities in cancer classification, subtyping, outcome prediction, and biomarker discovery, with improved generalization across institutions and tissue types compared to traditional task-specific models (94, 95). Importantly, they utilize transformer-based architectures (neural network designs originally developed for language understanding that excel at identifying long-range relationships and patterns) which enable scalable learning, enhanced robustness across different cohorts, and support for “few-shot” learning (adapting to new tasks with only a handful of examples) and even “zero-shot” inference (performing tasks they were never explicitly trained for) across diverse pathology applications (95).

**Table 1** summarizes the key pathology foundation models, their training data, architecture, and demonstrated capabilities. UNI, developed by Chen et al. (2024), was pretrained using DINOv2 self-supervised learning on over 100 million histopathology image patches from more than 100,000 whole slide images spanning 20 major tissue types, and demonstrated state-of-the-art performance across 34 clinical tasks including cancer subtyping, grading, and biomarker prediction (96). CONCH (CONtrastive learning from Captions for Histopathology), by Lu et al. (2024), is a vision-language foundation model trained on 1.17 million histopathology image-caption pairs, achieving state-of-the-art results across 14 benchmarks encompassing image classification, segmentation, captioning, and cross-modal retrieval tasks (97). Prov-GigaPath, developed by Xu et al. (2024), was pretrained on approximately 1.3 billion pathology image tiles derived from 171,189 WSIs across 31 tissue types, utilizing DINOv2 at the tile level followed by LongNet-based masked autoencoder pretraining at the slide level, and achieved significant improvements across 17 genomic prediction tasks and 9 cancer subtyping tasks (98). Virchow2, introduced by Zimmermann et al. (2024), was trained on 1.7-1.9 billion tiles from 3.1 million WSIs using DINOv2, representing one of the largest pathology datasets to date, and demonstrated the highest consistency across TCGA, CPTAC, and external benchmarking tasks in independent evaluations (99, 100). A recent independent clinical benchmark by Campanella et al. systematically compared these foundation models, finding that while most achieved similar performance for disease detection tasks, UNI and Prov-GigaPath consistently demonstrated superior performance in biomarker prediction and treatment outcome prediction tasks, though performance variability across external datasets underscores the ongoing challenge of ensuring generalizability (101).

**Table 1:** Key pathology foundation models.

Model	Training data	Architecture	Key capabilities	Notable performance
UNI (96)	100M+ patches, 100K+ WSIs, 20 tissue types	ViT-Large, DINOv2 self-supervised learning	Cancer classification, subtyping, biomarker prediction, few-shot learning	SOTA on 34 clinical tasks; top performer in biomarker and treatment outcome prediction
CONCH (97)	1.17M image-caption pairs	ViT + contrastive vision-language learning (CoCa)	Image classification, segmentation, captioning, image-text retrieval	SOTA across 14 benchmarks; superior for non-H&E stains (IHC, special stains)
Prov-GigaPath (98)	1.3B tiles, 171K WSIs, 31 tissue types	ViT + DINOv2 tile-level + LongNet slide-level MAE	WSI-level embeddings, pan-cancer subtyping, genomic prediction	Significant improvements across 17 genomic prediction and 9 cancer subtyping tasks
Virchow2 (99, 100)	1.7-1.9B tiles, 3.1M WSIs	ViT-Huge/Giant, DINOv2	Pan-cancer classification, grading, pathway activity prediction	Highest consistency across TCGA, CPTAC, and external benchmarking tasks

CONCH, CONtrastive learning from Captions for Histopathology; CPTAC, Clinical Proteomic Tumor Analysis Consortium; DINOv2, self-DIstillation with NO labels version 2; H&E, hematoxylin and eosin; IHC, immunohistochemistry; MAE, masked autoencoder; SOTA, state-of-the-art; TCGA, The Cancer Genome Atlas; ViT, Vision Transformer; WSI, whole slide image.

For practicing pathologists, the practical advantage of foundation models is that they could enable rapid deployment of AI tools for emerging biomarkers or rare diagnoses without waiting years for the accumulation of thousands of labeled training cases. However, this versatility also raises important questions about validation: if a model can perform tasks it wasn’t specifically trained for, how do we ensure it performs them safely and accurately?

### 4.2 Multimodal integration: beyond single data types

**Figure 1:**
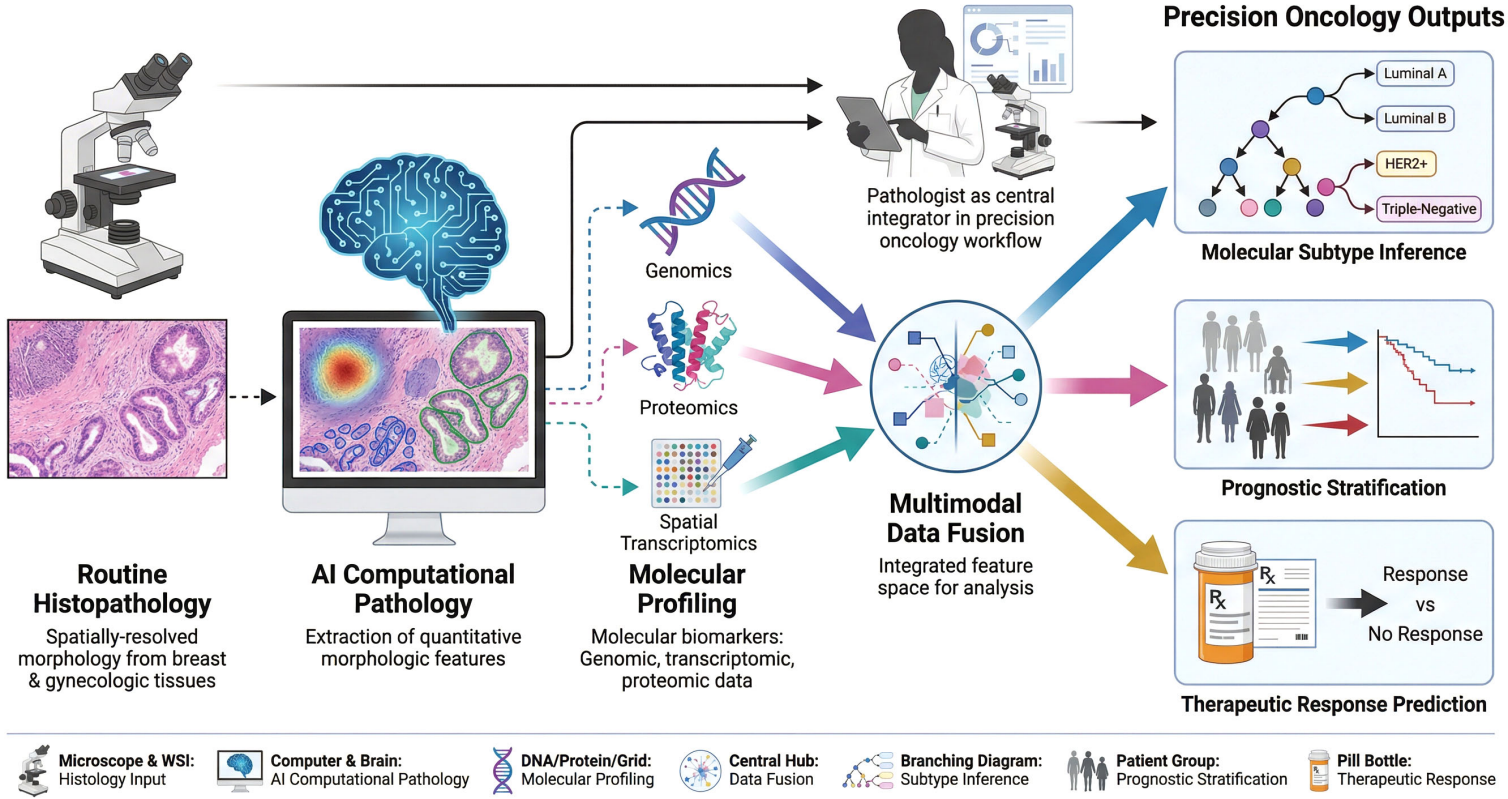
Multimodal integration framework in precision oncology. Schematic illustrating the convergence of routine histopathology, artificial intelligence (AI)-based computational pathology, and molecular profiling (genomics, proteomics, and spatial transcriptomics) into a unified multimodal data fusion platform. In this workflow, the pathologist serves as the central integrator of morphologic, molecular, and computational outputs. Integrated analysis enables clinically relevant precision oncology applications, including molecular subtype inference, prognostic stratification, and prediction of therapeutic response in breast and gynecologic cancers. Created with FigureLabs.

While early AI systems in pathology focused exclusively on analyzing histological images, emerging multimodal models integrate information from multiple data types (combining histopathology with genomics, transcriptomics, proteomics, and clinical data) to achieve more comprehensive tumor characterization than any single modality can provide (**Figure 1**). These advanced models incorporate cross-attention mechanisms, computational frameworks that enable bidirectional information flow between modalities, allowing histological features to inform genomic interpretation and vice versa (102, 103).

For example, the CATfusion model achieved superior performance for pan-cancer survival prediction by integrating mRNA sequencing, miRNA sequencing, copy number variation data, DNA methylation patterns, mutation profiles, and histopathological images through cross-attention transformers (102). This multimodal approach mirrors how pathologists intuitively integrate morphology with immunohistochemistry, molecular results, and clinical history, but does so at a scale and with a quantitative rigor that exceeds human capacity.

The clinical promise of multimodal AI lies in its ability to identify subtle correlations between tissue morphology and molecular alterations that are invisible to the human eye, potentially revealing new biomarkers or therapeutic targets. However, these complex models also raise concerns about interpretability: when a model integrates dozens of molecular and morphological features, understanding why it makes a particular prediction becomes increasingly challenging, which may limit clinical trust and regulatory acceptance.

### 4.3 AI assistants and conversational interfaces in pathology

The emergence of AI “copilots” such as PathChat and SmartPath represents an attempt to make AI more accessible and intuitive for pathologists through conversational interfaces that combine large language models with computer vision capabilities (94, 104). These systems aim to enable natural language queries about histopathology images (e.g., “What is the mitotic count in this field?” or “Are there features suggestive of lymphovascular invasion?”), automated report generation with structured synoptic elements, and interactive diagnostic assistance that responds to pathologists’ questions in real-time (94, 104).

While this vision is compelling, significant challenges remain. Most critically, current generative AI systems are prone to “hallucinations,” generating plausible-sounding but factually incorrect information with high confidence (94). In diagnostic pathology, where a single error can lead to incorrect treatment decisions, this risk is unacceptable. A generative AI might, for instance, confidently describe features that are not present in the image or cite nonexistent literature to support its interpretation.

Additionally, concerns regarding model explainability are particularly acute for these complex systems: when an AI copilot makes a diagnostic suggestion, pathologists need to understand the reasoning behind it to appropriately calibrate their trust. Regulatory frameworks for generative AI in pathology remain underdeveloped, requiring novel approaches to validation, oversight, and post-deployment monitoring that go beyond traditional software regulation. Before these tools can be safely deployed in clinical practice, reliable mechanisms for detecting and preventing hallucinations, validating outputs against ground truth, and monitoring real-world performance must be established (94).

### 4.4 Intraoperative AI: real-time decision support

An emerging application of AI in pathology is real-time intraoperative decision support using rapid analysis of frozen sections to guide surgical decision-making (20, 105, 106). During cancer surgery, pathologists often perform frozen section analysis to inform critical decisions such as margin assessment, lymph node evaluation, and rapid tumor classification. AI systems designed for this application must meet stringent requirements: ultra-fast inference times (ideally seconds, not minutes), reliable performance despite frozen section artifacts, and seamless integration with surgical workflows to deliver results when they are needed (20). However, the high-stakes, time-pressured intraoperative environment demands even higher standards of reliability than those required in routine diagnostic applications. False negatives could lead to incomplete cancer resection requiring reoperation, while false positives could result in unnecessary tissue removal and functional impairment. Prospective validation in real surgical settings, with backup pathologist review, will be essential before widespread adoption. If successfully implemented, intraoperative AI could reduce surgical time, improve margin clearance rates, and potentially reduce the need for second surgeries in selected cases.

### 4.5 Integration with liquid biopsy: bridging tissue and blood-based diagnostics

An exciting future direction involves integrating digital pathology AI with liquid biopsy data to create a more complete picture of tumor biology that combines spatial information from tissue with temporal dynamics from serial blood sampling (107–110). Liquid biopsy technologies analyze circulating tumor DNA (ctDNA), circulating tumor cells (CTCs), extracellular vesicles, and tumor-educated platelets to provide real-time insights into tumor burden, treatment response, and emergence of resistance mechanisms through minimally invasive blood draws (107–110).

In breast cancer, ctDNA detection and enumeration of CTCs (≥5 CTCs per 7.5 mL of blood in metastatic disease) correlate with worse prognosis and provide prognostic information and track treatment efficacy. Specific genetic mutations and methylation signatures detected in ctDNA have applications in early cancer screening, minimal residual disease monitoring after treatment, and tracking the evolution of drug resistance mechanisms in real-time (109, 111). The integration of AI-predicted molecular features from primary tumor histology with longitudinal liquid biopsy data could enable dynamic risk stratification and early detection of disease recurrence before it becomes visible on imaging, potentially allowing earlier therapeutic intervention (112, 113).

In gynecologic malignancies, liquid biopsy has demonstrated particular promise for detecting molecular alterations in endometrial cancer (including MMR deficiency and *POLE* mutations that guide treatment decisions), monitoring platinum response in ovarian cancer, and identifying HPV-associated molecular pathways in cervical cancer (107, 108, 114). The combination of AI-derived spatial features from primary tumor tissue with serial ctDNA monitoring may provide complementary information about tumor heterogeneity and clonal evolution that neither modality captures independently (115–117). For example, AI analysis of the primary tumor might reveal spatial patterns suggesting high heterogeneity, prompting more intensive liquid biopsy monitoring for the emergence of resistant clones.

### 4.6 Toward comprehensive multimodal tumor characterization

Multimodal integration frameworks that combine histopathology-derived AI predictions, spatial molecular profiling of tissue samples, and liquid biopsy analytes represent the next frontier in precision oncology (117–119). This comprehensive approach could potentially capture both the spatial organization of the TME at a single time point (through tissue analysis) and the temporal dynamics of tumor evolution over months to years (through serial blood sampling), providing a four-dimensional view of cancer biology.

For instance, AI analysis of a resected endometrial cancer might identify high-risk spatial features that trigger closer ctDNA monitoring. Rising ctDNA levels during surveillance could prompt earlier imaging or therapeutic intervention before clinical recurrence. In ovarian cancer, integration of primary tumor spatial features with longitudinal ctDNA and CA-125 trends might improve the prediction of platinum resistance and guide the selection of alternative therapies.

However, significant challenges must be addressed before this vision becomes a clinical reality. Standardization of liquid biopsy techniques remains incomplete, with variability in blood collection methods, processing protocols, and analytical platforms affecting results (108, 115, 118). Interpretation of liquid biopsy findings requires expertise that is still concentrated in specialized centers, and optimal thresholds for clinical action (e.g., what level of ctDNA increase should trigger intervention) remain undefined for most cancer types. Integration of liquid biopsy data with tissue-based diagnostics requires sophisticated bioinformatics infrastructure and data management systems that few institutions currently possess (107).

For pathologists, these emerging technologies suggest a future role that is less focused on routine pattern-recognition tasks (which AI may increasingly handle) and more on the complex integration of multimodal data, quality oversight of AI systems, correlation of findings with clinical context, and communication of nuanced diagnostic interpretations to clinical teams. Rather than replacing pathologists, these technologies are more likely to fundamentally transform the nature of pathology practice, requiring new skills in AI interpretation, multimodal data integration, and collaborative decision-making. Embracing these changes while maintaining the critical judgment and contextual expertise that define pathology will be essential as the field evolves.

## 5 Clinical translation: from promise to practice

The translation of AI from research innovation to routine clinical use in pathology faces a complex landscape of regulatory, technical, economic, and human factors challenges. Understanding these barriers is essential for pathologists who will increasingly work alongside AI systems in their practice.

### 5.1 Regulatory pathways and evolving frameworks

The U.S. FDA’s approval of WSI systems for primary diagnosis marked a critical turning point, establishing digital pathology as a legitimate diagnostic modality and paving the way for AI applications (13, 17, 120). The FDA regulates AI-based pathology tools through the Software as a Medical Device (SaMD) framework, with most applications currently cleared via the 510(k) pathway, which requires demonstration of “substantial equivalence” to existing predicate devices but does not mandate comprehensive clinical trials proving improved patient outcomes (13).

Alternative pathways include the *De Novo* route for novel low- to moderate-risk devices without suitable predicates, and Premarket Approval (PMA) for the highest-risk applications requiring rigorous clinical evidence of safety and effectiveness (13). However, the current regulatory framework faces unique challenges with AI algorithms, particularly those designed to continuously learn and adapt from new data. A locked algorithm (fixed after initial training) remains static and can be validated like traditional software, whereas a continuous learning algorithm evolves over time and may become substantially different from what regulators initially reviewed.

To address this, the FDA has proposed innovative approaches, including the Software Pre-Certification (Pre-Cert) Pilot Program, which focuses on evaluating the organization developing the software rather than just the product itself, and a Total Product Lifecycle regulatory approach emphasizing ongoing real-world performance monitoring rather than relying solely on pre-market evaluation. International harmonization efforts through the International Medical Device Regulators Forum aim to establish consistent global standards, though significant variability in regulatory requirements across countries currently complicates international deployment (13, 16, 17, 121, 122).

For pathologists, the practical implication is that regulatory approval, while necessary, is not sufficient proof that an AI tool will work well in their specific practice setting. Even FDA-cleared tools require local validation to ensure performance in each institution’s unique environment.

### 5.2 Clinical validation: beyond algorithm performance

The College of American Pathologists (CAP) distinguishes between clinical verification (technical assessment using curated datasets in controlled conditions) and clinical validation (end-to-end testing in real clinical production environments using actual patient specimens) (123). While publications often report impressive performance metrics from verification studies, these may not reflect real-world clinical utility.

Clinical validation must establish performance characteristics, including accuracy, precision, analytical sensitivity, analytical specificity, and reportable range, in the specific deployment environment. Critical considerations include ensuring test datasets represent the deployment site’s patient demographics, pre-analytic variables (tissue processing, fixation times, staining protocols), and technical infrastructure (scanner type, image compression), as performance can vary substantially across institutions (123, 124).

External validation on independent datasets from multiple institutions is essential to assess generalizability, with particular attention to potential performance degradation in underrepresented populations (124, 125). A model trained primarily on academic medical center data may perform poorly at community hospitals with different patient populations, disease prevalence, or tissue processing workflows. Recent studies suggest that community hospitals may benefit more from models pre-trained on community hospital data, rather than if diverse academic training data always produces better generalization (126).

The domain shift problem, where models show excellent performance on internal validation but reduced accuracy when deployed elsewhere, represents one of the most significant barriers to widespread AI adoption (124, 127, 128). This degradation reflects differences in patient demographics, disease prevalence, tissue processing protocols, scanning equipment, and staining variability that training datasets fail to adequately capture. Until AI models demonstrate robust cross-institutional performance or institutions develop efficient workflows for local customization and validation, each deployment site must budget time and resources for validation studies before clinical implementation.

An emerging model for clinical validation is “silent prospective” deployment, in which AI models are run prospectively on clinical cases in the background without influencing diagnostic decisions, allowing institutions to accumulate real-world performance data on their own patient populations and workflow conditions before formal clinical integration. Campanella et al. demonstrated this approach for lung cancer biomarker detection using a fine-tuned pathology foundation model, providing a practical framework for bridging the gap between retrospective validation studies and full clinical deployment (23). This approach allows institutions to assess model performance on local patient populations, identify potential failure modes, and build clinician confidence before AI outputs are used for patient care decisions.

### 5.3 Human-AI interaction: team performance matters

Standalone evaluations of AI algorithm performance provide an incomplete assessment of clinical utility. What matters is not how accurately AI performs in isolation, but how well the human-AI team performs when pathologists use AI assistance in clinical practice (123). Reader studies comparing pathologist accuracy with and without AI assistance provide critical insights, often revealing complex interaction effects.

Studies have demonstrated that AI assistance can improve diagnostic accuracy, but also introduces risks of automation bias; i.e., the tendency to over-rely on algorithmic outputs and fail to critically evaluate AI predictions, particularly when pressed for time or fatigued (123, 129). Presentation factors matter: the complexity of the user interface, display features, explainability of model outputs, and information density all influence user stress levels and diagnostic reliability (123).

Best practices for implementation include developing model fact sheets that communicate essential information to clinical users: intended use cases, warnings about inappropriate applications, performance characteristics across different subgroups, training data sources, known limitations, and criteria for discontinuation (105, 127). Transparency builds appropriate calibration of trust. Pathologists should neither blindly accept nor reflexively dismiss AI outputs, but rather critically integrate them based on an understanding of model strengths and weaknesses (125–127, 130, 131).

International surveys reveal substantial variation in pathologists’ opinions regarding AI adoption, with a lack of consensus on sufficient validation standards, preferred AI roles in workflows, and timing of AI introduction in training (131). Pathologists consistently emphasize that trust in AI systems develops through evaluation opportunities, hands-on experience, and familiarity with performance characteristics rather than through vendor claims or regulatory approval alone (132, 133). Importantly, whether AI should work autonomously or require pathologist review varies by specific task and clinical context. Pathologists generally accept automation for well-defined, routine tasks but desire human oversight for complex, high-stakes decisions.

### 5.4 Infrastructure and technical implementation

Successful AI implementation requires a comprehensive digital infrastructure that many pathology laboratories currently lack. Requirements include:

- WSI systems: high-throughput scanners capable of digitizing sufficient volume for routine practice
- Storage infrastructure: massive capacity for WSI data (a single slide can generate 1 to 5 GB of image data; a mid-sized laboratory might generate terabytes annually)
- Computing resources: high-performance servers for running AI algorithms, particularly for computationally intensive deep learning models
- Network bandwidth: robust connectivity for rapid image transfer between storage, analysis, and viewing systems
- Integration with laboratory information systems: seamless data flow to avoid manual data entry and enable automated workflow

The initial capital investment for comprehensive digital pathology infrastructure can exceed several million dollars for large institutions, with ongoing costs for maintenance, software licensing, storage expansion, and system upgrades. These costs pose challenges for community hospitals and resource-limited settings, potentially creating a “digital divide” where sophisticated AI tools are available only at well-resourced academic centers.

Data standardization remains a critical technical challenge (124, 127). AI models trained on images from a single scanner or staining protocol may exhibit substantial performance degradation when deployed on different equipment or in laboratories with different tissue-processing procedures. Color variation from different staining batches, image compression artifacts, focus quality differences, and even slide label placement can affect model performance. This lack of standardization necessitates either extensive validation for each scanner-stain combination or development of more robust, invariant algorithms, both resource-intensive approaches.

### 5.5 Addressing bias and ensuring equity

AI models may perpetuate or exacerbate healthcare disparities if not carefully developed and validated. Algorithmic bias can arise from multiple sources:

- Data bias: training datasets that underrepresent certain populations (racial/ethnic minorities, specific geographic regions, rare disease variants) lead to models with reduced performance for these groups
- Development bias: design choices during algorithm development, including feature selection and optimization objectives, may inadvertently favor majority populations
- Interaction bias: differential trust in or interpretation of AI outputs across demographic groups can lead to disparate clinical impact even when model performance is equivalent

The consequences are serious; AI systems that perform poorly for underrepresented populations may lead to delayed diagnosis, inappropriate treatment recommendations, or worse outcomes for already marginalized groups. Ensuring fairness requires deliberate efforts to include diverse populations in training datasets, to validate performance across demographic subgroups, to monitor for disparate impact during clinical deployment, and to establish governance frameworks that prioritize equity.

Transfer learning studies have revealed counterintuitive findings: models may not generalize well even within the same country if training and deployment sites serve different populations. A model trained predominantly on specimens from one racial/ethnic group may show degraded performance on others, even for seemingly objective tasks like tumor detection. This underscores the importance of demographic diversity in training data and explicit validation across population subgroups.

### 5.6 Explainability and the “Black Box” problem

Deep learning models typically function as “black boxes”; they produce predictions but offer limited insight into their reasoning process (129, 134, 135). This opacity creates challenges for clinical trust, regulatory approval, and error analysis. Various explainability methods have been developed, including attention maps (highlighting image regions the model focused on), saliency visualizations (showing which pixels most influenced the decision), and concept-based explanations (identifying high-level features the model learned). However, these techniques have significant limitations; they often fail to capture the full complexity of neural network reasoning, may themselves be misleading or cherry-picked, and generally provide *post-hoc* rationalizations rather than true mechanistic understanding (134, 136).

For clinical deployment, the level of explainability required likely depends on the application’s risk level and the availability of pathologist oversight. Fully automated tasks with well-defined scope and low error consequences may require less explainability than AI systems informing high-stakes treatment decisions. Regardless, pathologists need sufficient insight into model behavior to appropriately calibrate trust and identify when AI assistance should be accepted versus questioned.

### 5.7 Data privacy, ownership, and governance

The application of AI in pathology raises fundamental privacy and governance concerns (134, 136, 137). Pathology images, particularly when linked with molecular profiles, clinical data, and demographic information, contain rich personal health information. The potential for re-identification of patients from ostensibly de-identified images (especially when combined with other data sources) requires complex governance frameworks (135).

Data fusion enabled by computational pathology challenges traditional bioethics principles. Combining histological images with genomic data, radiological findings, and electronic health records creates comprehensive patient profiles with powerful research and clinical applications, but also substantial privacy risks. Traditional data ethics emphasizes purpose limitation (using data only for its original collection purpose) and data segregation (keeping different data types separate), while AI development demands large, integrated datasets. Navigating this tension requires updated governance frameworks, sophisticated consent processes that address data integration, and technical safeguards against unauthorized access or re-identification (135).

Data ownership questions remain unresolved: Who owns the AI-generated predictions? If a model identifies a prognostic feature not mentioned in the pathologist’s report, does the patient have a right to that information? Are institutions obligated to re-analyze archived cases when improved AI tools become available? These questions have legal, ethical, and practical implications that the pathology community is still working to address.

### 5.8 Accountability and liability

When AI systems contribute to diagnostic errors, accountability becomes complex. Potential responsible parties include algorithm developers, deploying institutions, individual pathologists, and regulatory agencies. Current liability frameworks, designed for traditional medical devices and human decision-makers, struggle to address AI systems that learn from data, operate probabilistically, and may fail in unpredictable ways. Establishing appropriate accountability that incentivizes safety while enabling innovation represents a critical challenge requiring collaboration among legal experts, regulators, healthcare institutions, and the pathology community (129). The practical implication for pathologists is that legal responsibility for diagnoses remains with the signing pathologist, regardless of AI assistance. AI tools do not currently share or assume liability; they augment pathologists’ capabilities but do not absolve pathologists of diagnostic responsibility.

### 5.9 Economic considerations and reimbursement

Despite technical feasibility, widespread AI adoption depends on economic viability (138). Current pathology reimbursement models typically do not account for the added value of AI-assisted diagnosis or the costs of implementing and maintaining AI systems (139–141). Without appropriate reimbursement, the substantial infrastructure, licensing, and operational costs may not be offset by revenue, limiting adoption, particularly in community practice settings.

Economic evaluations of AI healthcare interventions generally report favorable cost-effectiveness, with most published studies concluding that AI strategies are cost-effective, cost-beneficial, or cost-saving compared to standard approaches (138). However, methodological heterogeneity, incomplete cost reporting (often excluding implementation and training costs), and likely publication bias (positive studies are more likely to be published) constrain the reliability of this evidence (138, 139).

AI applications in pathology demonstrate potential for cost savings through increased efficiency, reduced turnaround times, decreased need for repeat testing, and improved diagnostic accuracy that leads to more appropriate treatment (140, 142). By automating routine tasks and standardizing subjective assessments, AI could enable pathologists to serve more patients while maintaining quality (140, 142). However, upfront costs must be considered: scanner acquisition (USD 200,000-500,000+ per high-throughput unit), digital infrastructure (USD 500,000-2,000,000 for mid-sized laboratories), software licensing (variable models from per-case fees to annual subscriptions), maintenance, and ongoing validation (138, 139).

Payers require evidence of clinical utility (demonstration that AI improves patient outcomes, not just diagnostic accuracy) before establishing reimbursement policies. Diagnostic accuracy is necessary but insufficient; what matters is whether AI leads to earlier cancer detection, more appropriate treatment selection, reduced diagnostic errors, or other outcomes that benefit patients. This evidence gap represents a significant barrier, as generating this data requires expensive prospective studies.

Standardized economic evaluation frameworks that incorporate comprehensive cost structures and account for AI’s unique features (scalability, dynamic evolution, potential for rapid improvement) are urgently needed (138). Current Procedural Terminology (CPT) codes specifically addressing AI-assisted pathology services are limited, creating uncertainty about billing and reimbursement (141). Until these issues are resolved, sustainable business models for AI in pathology remain challenging, particularly for commercial developers.

### 5.10 Ensuring long-term performance: model monitoring and maintenance

Even well-validated AI systems can experience performance degradation over time due to model drift, in which changes in technology, clinical practice, patient populations, or disease patterns cause the clinical environment to evolve beyond the conditions represented in the training data (143, 144). Examples include new staining protocols altering tissue appearance, scanner upgrades changing image characteristics, shifting patient demographics, emerging disease variants, and changes in pre-analytic workflows.

Maintaining algorithm accuracy requires continuous monitoring of real-world performance and periodic retraining or recalibration (resource-intensive activities that institutions must budget for over the long term). Regulatory frameworks are evolving to address these requirements, with increasing emphasis on post-market surveillance and ongoing quality assurance rather than one-time pre-market approval.

## 6 Preparing the pathology workforce

The integration of AI into pathology practice necessitates fundamental changes in pathologists’ competencies, extending beyond traditional morphological expertise to encompass technology evaluation, implementation, and critical appraisal of algorithmic outputs (143, 144).

### 6.1 New competencies for pathologists

While most current entrustable professional activities (EPAs) and competencies remain relevant, new capabilities are essential:

- Understanding AI fundamentals: basic knowledge of machine learning concepts, deep learning architectures, and how models are trained and validated
- Data literacy: interpreting performance metrics (sensitivity, specificity, AUC, confidence intervals, positive/negative predictive values), understanding validation methodologies, and recognizing sources of bias and error
- Critical evaluation: assessing published AI studies, identifying methodological limitations, and determining applicability to local practice
- Implementation and validation: participating in algorithm selection, local validation studies, and ongoing quality assurance
- Human-AI interaction: integrating AI outputs into diagnostic workflows, recognizing automation bias, maintaining appropriate skepticism, and knowing when to override AI suggestions
- Ethical awareness: understanding privacy implications, fairness concerns, and accountability issues in AI deployment

### 6.2 Educational integration

Exposure to digital pathology and AI should begin in medical school and continue through residency training (144, 145). However, current Liaison Committee on Medical Education (LCME) and Accreditation Council for Graduate Medical Education (ACGME) frameworks do not mandate AI literacy, creating preparation gaps (144). Proposed curricula should address:

- Foundational concepts: machine learning basics, statistical principles, digital image analysis
- Hands-on experience: using digital pathology platforms, working with AI-assisted diagnostic tools, participating in validation studies, learning annotation methodologies for model development
- Critical appraisal: evaluating AI literature, understanding study designs and performance metrics, identifying algorithmic bias
- Practical integration: incorporating AI into diagnostic workflows, communicating AI-assisted findings to clinicians
- Ethics and governance: privacy protection, algorithmic fairness, liability considerations

### 6.3 Faculty development and institutional support

Faculty development represents a critical bottleneck (145, 146). Many current pathologists lack formal training in AI, digital pathology, or computational methods, limiting their ability to teach these topics effectively or to supervise trainees. Institutions must invest in:

- Faculty education programs in AI fundamentals and digital pathology
- Protected time for curriculum development
- Collaboration opportunities with computer scientists, bioinformaticians, and data scientists
- Resources for establishing digital pathology teaching collections
- Support for faculty research in AI development and validation

Foundation models and generative AI tools may enhance self-directed learning by providing interactive, personalized educational experiences (144, 147). However, these require faculty oversight, instruction in critical appraisal, and safeguards against inaccuracy and overconfidence in AI-generated content.

### 6.4 The evolving role of pathologists

Rather than replacing pathologists, AI is likely to transform the nature of pathology practice. The future pathologist’s role may emphasize:

- Complex diagnostic integration: synthesizing information from multiple sources (morphology, molecular data, AI predictions, clinical context) to reach nuanced diagnostic conclusions
- Quality oversight: ensuring AI systems perform reliably, identifying failures, and maintaining diagnostic accuracy
- Specialized expertise: focusing on challenging cases, rare entities, and situations requiring expert judgment while AI handles routine tasks
- Collaborative medicine: communicating complex, multimodal diagnostic information to clinical teams and patients
- Innovation and research: participating in AI development, validation, and continuous improvement

## 7 Conclusions

The convergence of digital pathology, molecular profiling, and AI represents a fundamental shift in breast and gynecologic oncology, moving from parallel diagnostic streams toward integrated, multimodal platforms that extract biological meaning from routine histology while contextualizing molecular alterations within tissue architecture. This integration promises more efficient, reproducible, and biologically informed diagnostics that enhance precision oncology and improve patient outcomes.

Key achievements over the past decade include FDA approval of WSI for primary diagnosis, development of AI models that predict molecular biomarkers from H&E images with clinically relevant accuracy, successful molecular classification of endometrial cancer subtypes from histology, and the emergence of spatial profiling technologies that illuminate tumor-microenvironment interactions influencing therapeutic response. Foundation models trained on millions of histopathology images demonstrate superior generalization and enable few-shot learning, potentially democratizing access to expert-level pathology interpretation.

Critical challenges remain, including ensuring algorithm generalizability across diverse populations and practice settings, establishing standardized validation frameworks and regulatory pathways for adaptive algorithms, developing sustainable reimbursement models that recognize AI’s clinical value, and addressing ethical concerns regarding bias and equity.
